# The role of interleukin-20 in liver disease: Functions, mechanisms and clinical applications

**DOI:** 10.1016/j.heliyon.2024.e29853

**Published:** 2024-04-17

**Authors:** Kun Wang, He-Qin Zhan, Ying Hu, Zhan-Yuan Yuan, Jun-Fa Yang, Da-Shuai Yang, Liang-Song Tao, Tao Xu

**Affiliations:** a School of Clinical Medicine, Anhui Medical University, Hefei, 230032, China; b Department of Pathology, School of Basic Medical Sciences, Anhui Medical University, Hefei, 230032, China; c Inflammation and Immune Mediated Diseases Laboratory of Anhui Province, Anhui Institute of Innovative Drugs, School of Pharmacy, Anhui Medical University, Hefei, 230032, China; d Institute for Liver Diseases of Anhui Medical University, Anhui Medical University, Hefei, 230032, China; e Department of Plastic Surgery, The Second Affiliated Hospital of Anhui Medical University, Hefei, Anhui, 230032, China; f Department of orthopedics, Anhui Children's Hospital, Hefei, Anhui, 230032, China

**Keywords:** Inflammation, Receptor, Antibody, Hepatic stellate cell, Hepatocellular carcinoma

## Abstract

Liver disease is a severe public health concern worldwide. There is a close relationship between the liver and cytokines, and liver inflammation from a variety of causes leads to the release and activation of cytokines. The functions of cytokines are complex and variable, and are closely related to their cellular origin, target molecules and mode of action. Interleukin (IL)-20 has been studied as a pro-inflammatory cytokine that is expressed and regulated in some diseases. Furthermore, accumulating evidences has shown that IL-20 is highly expressed in clinical samples from patients with liver disease, promoting the production of pro-inflammatory molecules involved in liver disease progression, and antagonists of IL-20 can effectively inhibit liver injury and produce protective effects. This review highlights the potential of targeting IL-20 in liver diseases, elucidates the potential mechanisms of IL-20 inducing liver injury, and suggests multiple viable strategies to mitigate the pro-inflammatory response to IL-20. Genomic CRISPR/Cas9-based screens may be a feasible way to further explore the signaling pathways and regulation of IL-20 in liver diseases. Nanovector systems targeting IL-20 offer new possibilities for the treatment and prevention of liver diseases.

## 1 Introduction

Liver disease, a global public health concern in the 21st century, is a pathological condition that affects and damages the liver. The most frequently discussed liver diseases include non-alcoholic fatty liver disease (NAFLD), alcohol-related liver disease, toxic liver injury, hepatitis B virus (HBV), hepatitis C virus (HCV) and hepatocellular carcinoma (HCC) [1]. Liver disease was reported to have caused 287,000 deaths in Europe in 2019, with 63,500 attributed to primary liver cancer [2]. Various liver diseases and conditions can cause damage to healthy liver cells, resulting in inflammation and cellular death [3]. Over time, the persistent effects of inflammation during cellular repair can lead to scarring and more serious complications. Moreover, the etiopathogenesis of most liver diseases involves the activation of innate immune cell-driven and pro-inflammatory cascades and cycles [4]. Therefore, focusing on the mechanisms linking inflammation and liver disease will help elucidate the mechanisms of liver injury and develop new clinical drug targets.

The IL-20 subfamily's IL-20 cytokine is produced by monocytes, fibroblasts, epithelial cells, granulocytes, and dendritic cells and mediates signaling communication between hepatic epithelial cells and leukocytes. It plays a functional role in the inflammatory pathology of the liver [5]. IL-20, which was first identified in 2001, appears in many genome scan studies and is a subject of interest [6]. In recent years, several studies have demonstrated its involvement in clinical applications related to rheumatoid arthritis, atherosclerosis, stroke, psoriasis, and osteoporosis [7]. Nevertheless, research on IL-20's pathophysiological role in liver injury is limited, and its function remains to be defined.

**Fig. 1:**
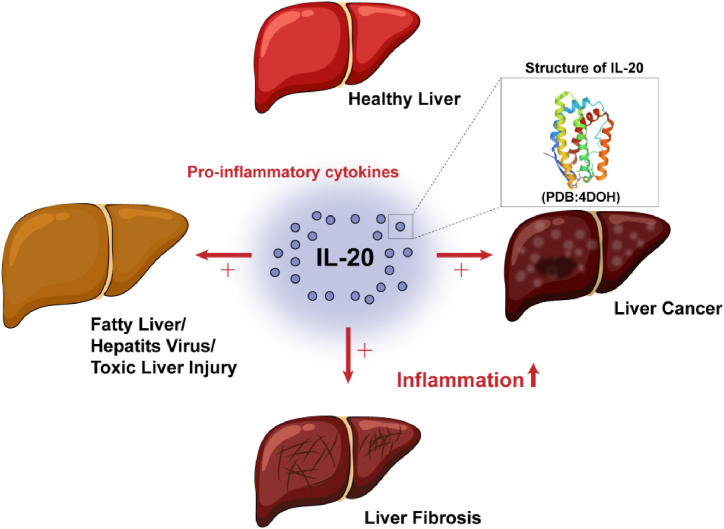
IL-20 acts as a pro-inflammatory cytokine and is involved in all progressions of liver diseases. Full-length human IL-20 structure (from protein data bank: https://www.rcsb.org/, PDB ID: 4DOH).

In this review, we summarize the main molecular features and biological functions of IL-20 and highlight the specific regulatory mechanisms and clinical applications that provide a basis for understanding the relationship between IL-20 and various types of liver disease (Fig. 1). As the mechanism of action of IL-20 continues to be elucidated, it is expected to be applied as a therapeutic target for the treatment of liver diseases.

## 2 Overview of IL-20 cytokine

The IL-20 subfamily belongs to the IL-10 family, which includes five members IL-19, IL-20, IL-22, IL-24, and IL-26. They are grouped together based on the remarkable similarity between their receptors and their target cells [8]. Monocytes, lymphocytes, natural killer cells, macrophages, epithelial cells, and fibroblasts are the primary cellular sources of these members [9,10]. In 2001, IL-20 was first identified in human keratinocytes using an EST database and a structure-based algorithm [11]. The gene encoding human IL-20 is located on chromosome 1 (1q32) [12]. Different cytokines of the IL-20 subfamily exert specific actions rather than broad anti-inflammatory effects [13]. Unlike IL-10, IL-20 may act as a pro-inflammatory cytokine in various diseases. For instance, it affects keratinocyte proliferation, leading to psoriasis. Additionally, it induces tumor necrosis factor (TNF)-α and IL-6 by monocytes and stimulates the expression of growth factors in CD8^+^ T cells [14]. Besides, it can exacerbate hepatocyte damage and aggravate acute hepatitis and bacterial infection by upregulating the expression of chemokines [10]. The interactions and synergistic effects of IL-20 with other members of the IL-20 subfamily are frequently discussed in research, and studying them together can help provide a more complete understanding of IL-20's role in physiologic and pathologic conditions. For example, it has demonstrated that the liver is a potential target of IL-19, IL-20, and IL-24 in the acute phase response [15]. Possible therapeutic approaches for the treatment of ALD could be the downregulation of IL-20 and the upregulation of IL-22 ratios through acquired epigenetic mechanisms [16]. Table 1 lists the roles of IL-20 subfamily members in liver diseases. Obviously, the functional role of IL-20 in liver diseases goes far beyond this and will be discussed later.

**Table 1:** Roles of IL-20 subfamily members in liver diseases.

IL-20 subfamily	Liver diseases	Functional role	[Ref]
IL-19	NAFLD	Inhibits lipid metabolism, such as triglyceride biosynthesis	[90]
IL-20	NAFLD	Promotes inflammation and exacerbates NAFLD	[21]
	HBV	Influences the outcome of HBV infection	[26]
	LF	Facilitates the value-added and migration of HSCs	[30]
	HCC	Upregulate the expression of cyclin D1, MMP-13 and TNF-α	[39]
IL-22	ALD	Improves ALD, liver damage and liver oxidative stress	[91]
	NAFLD	Reduces the release of inflammatory extracellular vesicles from hepatocytes	[20]
	HBV	Recruits Th17 cells and exacerbates inflammation	[92]
	HCV	Increases hepatocyte proliferation and inhibits apoptosis	[93]
	LF	Reduces α-smooth muscle actin expression in HSCs	[94]
	HCC	Promotes tumor growth	[95]
IL-24	HBV	Inhibits Th17 cell responses	[96]
	LF	Prevents the development of liver fibrosis	[44]
	HCC	Inhibits subcutaneous tumor growth	[97]
IL-26	HBV	Involves chronic inflammation	[98]
	HCV	Blocks the replication of viruses	[99]
	LF	Regulates the activation of NKs and reduces HSCs	[100]
	HCC	Inhibits the migration and invasion ability of HCC cells	[101]

NAFLD, Non-alcoholic fatty liver disease; HBV, Hepatitis B virus; LF, liver fibrosis; HCC, Hepatocellular carcinoma; MMP-13, Matrix metalloproteinase-13; TNF-α, Tumor necrosis factor-α; ALD, Alcoholic liver disease; Th17, T helper cell 17; HSC, Hepatic stellate cell; NK, Natural killer cell.

## 3 Function role of IL-20 in live diseases

Liver can be regarded as an immune organ and liver inflammation is the primary cause of liver injury. Inflammation stimulates the organism and produces a large number of cytokines and chemokines, which affect the synthesis of acute phase reactants in the liver. To better understand the crucial roles and connections between IL-20 expression and the emergence of liver disorders, we will briefly review the biological regulatory roles of IL-20 in various liver diseases.

### 3.1 Non-alcoholic fatty liver disease

NAFLD affects 25 % of individuals worldwide, and its incidence is increasing at an annual rate of 1 % [17]. Without prompt treatment, it can lead to cirrhosis and HCC. NAFLD can be divided into two types: nonalcoholic steatohepatitis (NASH) and simple steatosis, which share the common feature of large accumulation of fat in hepatocytes. However, NASH is associated with more severe liver damage, including hepatocellular swelling, inflammatory cell aggregation, and fibrosis, than simple steatosis [18]. Although various NASH drugs based on new theories and targets have entered clinical studies, none have been approved for marketing because of the complexity of their pathophysiology and the significant heterogeneity of the disease phenotype [19]. Interleukins have immense therapeutic potential in the treatment of NASH. Recent studies have suggested that IL-22 alleviates inflammation triggered in a neutrophil-driven NASH model by regulating metallothionein expression in the liver and reducing the release of inflammatory extracellular vesicles [20]. Of note, IL-22 and IL-20 are both members of the IL-20 subfamily, but show diametrically opposed trends in the regulation of inflammation in NAFLD. Estep et al. demonstrated that IL-20 expression was increased in the liver tissues of NAFLD patients, negatively correlated with microRNAs (miRNAs) in the white adipose tissue of patients, and positively correlated with inflammatory markers and the severity of NAFLD. miRNAs are a class of non-coding RNAs that affect the biological behavior of cells. Reduced miRNA expression in adipose tissue may lead to increased levels of mRNA encoding soluble pro-inflammatory molecules that are released into the bloodstream and bind to receptors in the liver parenchyma. The levels of pro-inflammatory signals, apoptosis, and necrosis are elevated in the liver, and IL-20, a pro-inflammatory molecule, is released as a pro-inflammatory signal that affects liver tissue and is involved in the pathogenesis of NASH. Apart from this, IL-20 was upregulated in a consistent trend with alanine aminotransferase and aspartate aminotransferase levels, which are indicators of liver function, and their elevation suggests the potential presence of inflammation, necrosis, and hepatocyte toxicity in hepatocytes [21]. Using a multi-omics approach, Gaucher et al. revealed that multiple dysregulation markers of NASH contain IL-20 [22]. Hence, the rational inhibition of IL-20 expression in NAFLD and the development of associated medicines targeting IL-20 are essential for the investigation and prevention of NAFLD.

### 3.2 Hepatitis B virus

HBV is a contagious infection caused by hepatophilic DNA virus. Long-term viral infections can lead to severe hepatitis and HCC. According to WHO data, 1.5 million new infections occur every year, with 296 million people infected with HBV in 2019, resulting in 820,000 fatalities [23,24]. Contact with blood, saliva, and semen carrying HBV can lead to the transmission of the virus. Furthermore, HBV is a recessive virus that generates and secretes large amounts of viral antigens accompanied by immune dysfunction, triggering the recruitment of large amounts of intrahepatic cytokines and chemokines, as well as the aggregation of regulatory cells involved in this series of pathogenic events [25]. HBV infection can be classified into three stages. The second stage is the immune response stage, which is accompanied by necro-inflammatory activity in the liver tissue. Immunologically, this phase is characterized by the secretion of cytokines such as IL-20, which recruit other immune cells and trigger acute inflammation of the liver tissue along with increased fibrosis. The balance of the immune response is dysregulated owing to a sustained pro-inflammatory response. This explains why liver tissue lesions are usually accompanied by IL-20 overexpression. Moreover, the HBV genotype, HBV clearance, HBeAg seroconversion, and HBV-related cirrhosis were all closely correlated with IL-20 expression. Further evidence indicates that the outcome of HBV infection is influenced by single nucleotide polymorphism variants in IL-10 and IL-20 genes. In other words, host genetic variability of IL-20 affects the outcome of HBV infection [26]. In summary, it can be found that HBV infection of hepatocytes does not directly cause hepatocellular damage, but rather favors the induction of an intrahepatic adaptive immune response through the recruitment of cytokines and triggering liver inflammation, which in turn leads to the persistence of HBV infection. Therefore, it is necessary to investigate the role of IL-20 in the pathogenesis of HBV.

### 3.2 Liver fibrosis

The combined annual incidence of fibrosis-related diseases is approximately 4968 per 100,000 people [27]. Liver fibrosis is a reparative response of the body to liver inflammation or injury and is pathologically characterized by the excessive deposition of diffuse extracellular matrix (ECM) in the liver and the activation of hepatic stellate cells (HSCs) [28,29]. Due to the complexity of the various types of inflammation and its interaction of inflammation with metabolic and genetic factors during fibrosis, there is no universally accepted treatment to stop the progression of fibrosis. Therefore, identifying indicator markers of inflammatory pathways involved in the pathogenesis of fibrosis may be a therapeutic breakthrough [1]. The inflammatory environment in the body during hepatocellular injury in liver fibrosis induces hepatocytes or activated Kupffer cells to release IL-20 as an inflammatory mediator, which activates the transformation of quiescent HSCs into myofibroblasts and expression of α-smooth muscle actin (α-SMA) and pro-fibrotic genes. This is accompanied by a sustained upregulation of myofibroblast activity, which leads to pathologically excessive deposition of ECM proteins, causing scarring and tissue hardening and exacerbating the fibrotic process. Chiu et al. revealed by immunohistochemistry that IL-20 expression was not only increased but also co-stained with α-SMA in the liver tissues of patients with hepatic fibrosis, further illustrating the high expression of IL-20 in HSCs. Furthermore, Chiu et al. found by qRT-PCR that mRNA expression of the inflammatory cytokines TNF-α, transforming growth factor (TGF)-β1, and collagen type I (Col I) was significantly elevated in HSCs treated with IL-20. The expression of TNF-α, monocyte chemotactic protein-1, Col I, metalloproteinases (TIMP)-1, and TIMP-2 was significantly increased, while the expression of matrix metalloproteinases (MMP)-2 and MMP-12 was significantly decreased in the liver tissues of mice with long-term carbon tetrachloride (CCL_4_)-induced liver injury [30]. The results of the experiment clearly support that IL-20 activates quiescent HSCs and also acts on activated HSCs, upregulating the expression of the cyclin-dependent kinase (CDK) inhibitor p21^WAF1^ and TGF-β1. TGF-β1 mediated by p21^WAF1^ arrests cells in the G1/S phase and induces differentiation and apoptosis of hepatocytes. Importantly, increased IL-20 facilitates the value-added and migration of HSCs, disrupts the balance between MMPs and TIMPs, and induces massive deposition of the ECM component. In addition, IL-20 was found to induce liver fibrosis by upregulating Col I production in rat HSCs [31]. Taken together, the current work confirms that IL-20 is a pathogenic cytokine that is upregulated during liver fibrosis [28]. In a fibrotic environment, myofibroblasts are the main effector cells that produce the ECM. Inhibition of IL-20 reduces the conversion of HSCs into myofibroblasts and decreases the release of TGF-β1, halting the progression of fibrosis and restoring cellular function.

### 3.3 Hepatocellular carcinoma

One of the top three causes of cancer-related death in 46 nations is liver cancer. HCC accounts for 85–90 % of liver cancer cases. HCC can be induced by any factor that stimulates liver inflammation over a long period of time, and its causes include long-term alcohol consumption, HBV, HVC, NAFLD, and liver fibrosis [[32], [33], [34]]. In 2019, there were approximately 747,000 cases of HCC worldwide, representing a 70 % increase since 1990, with 480,000 deaths attributable to HCC [35,36]. When the disease leads to the death of a large number of patients, ways to improve the survival rate become a focus of attention. Survival is a key indicator for assessing the prognosis and treatment outcomes of patients with HCC [37]. Ding et al. compared IL-20 expression rates in 64 pairs of HCC and adjacent non-tumor tissues and found that IL-20 was not only up-regulated in most HCC regions, but also that patients with high IL-20 expression had poorer overall survival compared to patients with low IL-20 expression [38]. Meanwhile, Chiu et al. analyzed IL-20 mRNA expression in HCC tumor tissues from 26 patients using immunohistochemistry and qRT-PCR, and found that high IL-20 expression in HCC was associated with low overall survival [39]. These studies clearly indicate a relationship between IL-20 levels and poor prognosis. Furthermore, Cyclin D1 is a well-recognized marker of human malignancy, and uncontrolled activation of Cyclin D-CDK4/6 kinase drivers the development of many types of cancers [40]. To further characterize the link between IL-20 and HCC, Chiu et al. observed a positive correlation between IL-20 and Cyclin D1 mRNA expression in three different HCC cell lines [39]. Moreover, IL-20 may directly or indirectly cause overexpression of Cyclin D1, leading to uncontrolled cell cycle regulation, accelerated G1/S phase transition, abnormal cell proliferation, accumulation of mutant DNA, and promotion of tumorigenesis and development. In describing the role of the immune system in HCC, Giuseppe made it clear that IL-1, TNF-α, and IL-6 are involved in invasion and metastasis, whereas TGF-β and IL-20 reduce the anti-tumor immune response [41]. In brief, these findings imply that IL-20 is sensitive to the tumor microenvironment of HCC, negatively regulates the immune response, participates in HCC growth, and affects tumor aggressiveness. HCC is a complex regulatory system, and only a comprehensive understanding of the various HCC formation mechanisms can better maintain the dynamic balance of the tumor environment, thus delaying or even reversing HCC progression. Therefore, targeting IL-20 is beneficial for reshaping the tumor inflammatory microenvironment, establishing effective immune detection, and providing strong support for therapeutic strategies.

## 4 Regulatory mechanism of IL-20 in liver disease

The regulatory mechanism of IL-20 in liver disease is mediated by two main components: receptors and signaling pathways. Receptors are a class of molecules that conduct extracellular IL-20 signals and produce specific effects within the cell. Signaling refers to a series of intracellular proteins that regulate the activity of downstream proteins when IL-20 binds specifically to intracellular receptors as a ligand, thereby gradually amplifies the external signal, transmitting it, and ultimately producing an integrated series of cellular responses.

**Fig. 2:**
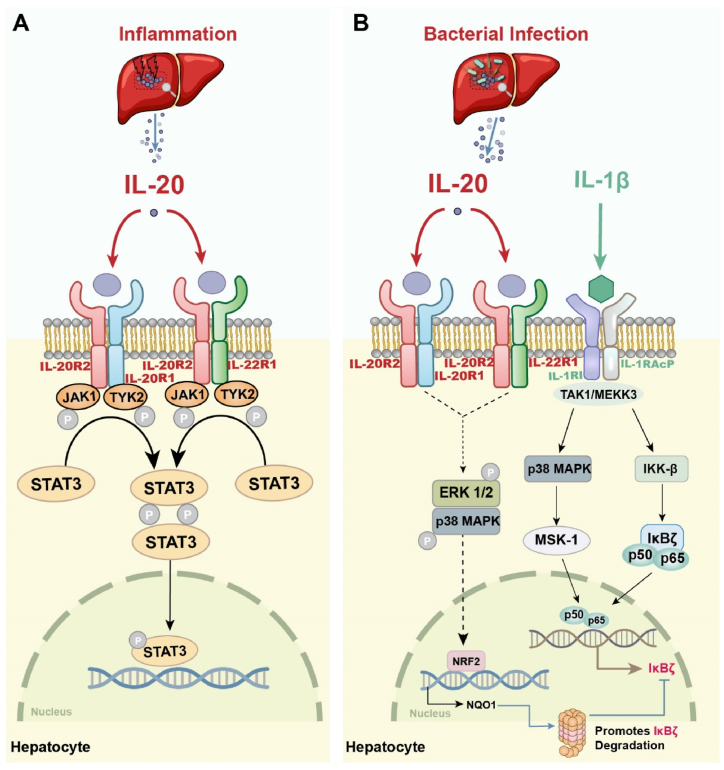
Regulatory mechanism of IL-20 in liver. (A) In the inflammatory environment of the liver, IL-20 phosphorylates and activates the heterodimeric receptor complexes IL-20R1/IL-20R2 and IL-22R1/IL-20R2. Upon binding to the receptors, IL-20 activates JAK1 and TYK2, recruits and phosphorylates the transcription factor STAT3, which then enters the nucleus in the form of a dimer and binds to the target genes, regulates the transcription of downstream genes. (B) During bacterial infection in liver, IL-20 activates the ERK1/2 and p38 MAPK signaling pathways through IL-20R2/IL-22R1, stimulates the expression of the NRF2 target enzyme NQO1, promotes IκBζ degradation, and induces liver injury. At the same time, the stimulated immune cells released large amounts of IL-1β, which further induced IL-20 gene expression through MAPK- and NF-κB-dependent mechanisms.

As previously described, members of the IL-20 subfamily share receptor subunits and signal through a common intracellular pathway Fig. 2. Receptor expression of IL-20 determines its biological properties [8]. IL-20 activates the heterodimeric receptor complexes of IL-20R1/IL-20R2 and IL-22R1/IL-20R2 [9,42,43]. It was shown that IL-20R2 was significantly upregulated in hepatocytes during the acute inflammatory response triggered by lipopolysaccharide and lipopolysaccharide-induced IL-20R2 expression enhanced hepatocyte sensitivity to cytokines [15]. Interestingly, Wang et al. analyzed the specificity of receptor expression of IL-20 and IL-24 in the hepatic fibrosis group and found that IL-20 and IL-24 utilize two identical receptor complexes but play opposite roles in hepatic fibrosis. Among these, IL-24 uses the IL-20R2/IL-22R1 receptor complex to protect hepatocytes. IL-24 does not interfere with protection even in the absence of IL-20R1, suggesting that IL-24 acts through IL-20R1-independent signaling. However, IL-20 tends to exert deleterious effects through the IL-20R1/IL-20R2 receptor complex, and IL-20R1 is required to induce activation of HSCs, which cannot be exerted in the absence of the IL-20R1 receptor. In conclusion, IL-20R1 receptors mediate the fibrotic process in the liver and stimulate the production of HSCs, and antagonism of the IL-20R1 receptors can effectively inhibit liver injury [44]. It is the different expression patterns of these receptors that distinguish IL-20 from other members. IL-20 has specific biological functions in the liver and is inextricably linked to the expression of its receptors.

After recognition by molecular pattern recognition receptors associated with liver damage, both immune and non-immune cell types exhibit abundant expression of IL-20 [45]. This leads to activation of JAK–STAT and NF-κB signaling pathways, subsequently influencing downstream transcriptional regulation. The JAK/STAT signaling pathway is thought to be a central communication node for cellular functions [46]. It is the IL-20 receptor that activates this signaling pathway and completes signaling (Fig. 2A). JAK/STAT mediates various downstream events including hepatocyte apoptosis, inflammatory cell infiltration, adipogenesis and tissue repair. Among them, JAK is a non-receptor type tyrosine protein kinase that includes four members: JAK1, JAK2, JAK3, and Tyk2. One cytokine can activate multiple intracellular JAKs or multiple cytokines can activate the same JAK simultaneously to exert biological effects. JAK binds to cell surface specific receptors and catalyzes the tyrosine phosphorylation and recruitment of STAT proteins. When tyrosine is phosphorylated, STATs dimerize and travel across the nuclear membrane to reach the nucleus where they bind to DNA regulatory elements and induce gene transcription [47]. The STAT family contains seven members, and different STATs have non-redundant biological effects, even though certain cellular factors can partially activate them at the same time. Among them, STAT3 is mainly involved in the immune response, cell growth, differentiation and apoptosis as well as in the negative regulation of tumorigenesis and metastasis [29]. Mechanistically, IL-20 receptors initiate signaling and recruitment of STAT3 proteins precisely by binding to JAK1 and TYK2, and phosphorylated STAT3 is translocated to the nucleus to modify gene transcription. Activation of STAT3 has been detected in many fibrotic tissues. Activation of STAT3 can play pro-inflammatory roles in the pathogenesis of liver fibrosis [48]. Several known STAT3 inhibitors, designed to directly target hyper-phosphorylated STAT3 in cells, have been used as chemical probes to certify the role of STAT3 in CCl4-induced liver fibrosis [49,50]. Approximately 60 % of phosphorylated STAT3 can be detected in human HCC tissues and sustained activation of STAT3 leads to the self-renewal of tumor stem cells and tumor susceptibility to recurrence, metastasis and progression. Hösel et al. detected both interleukin-dependent and -independent activation of STAT3 in hepatocytes replicating HBV, both in cell culture and in vivo [51]. Yang et al. verified that targeted blockade of STAT3 signaling effectively inhibited HBV replication [52]. In addition to STAT3, STAT5 can be activated by IL-20 but at a higher concentration [53,54]. Tritsaris et al. demonstrated that IL-20 stimulation of large vascular endothelial cells resulted in receptor-dependent activation of multiple intracellular signaling components, including increased JAK2/STAT5 phosphorylation [55]. STAT5 activation was significantly correlated with the aggressive behavior of HCC cells [56]. Unfortunately, the precise mechanism of IL-20 and STAT5 in liver diseases has not been definitively elucidated. Consequently, further in-depth exploration is required to elucidate the downstream pathways of STAT that are associated with IL-20. At this stage, the development of efficient and precise gene therapies targeting STAT is important for the treatment of liver diseases.

Furthermore, p38 mitogen-activated protein kinase (MAPK) affects IL-20 expression through the activation of mitogen and stress activated kinase 1 (MSK1) and NF-kB-driven IL-20 messenger RNA transcription (Fig. 2B), a mechanism of action that underscores the complex regulatory network involved in regulating IL-20 expression in the inflammatory response [57,58]. Kaymak et al. reported that IL-20 may be mediated by the MAPK and extracellular signal-regulated kinase (ERK) 1/2 pathways. Targeting the IL-20 signaling pathway using MAPK inhibitors may be a potential therapeutic strategy [59]. He et al. assessed that IL-20 can activate ERK/NRF2 and p38/NRF2 signaling pathways through IL-22R1/IL-20R2 receptors to stimulate the expression of the hepatic NRF2 targeting enzyme NAD(P)H: quinone oxidoreductase 1 (NQO1), and elevation of NQO1 results in the degradation of IκBζ in hepatocytes, which accelerates liver injury. Interestingly, IL-1β induces IκBζ expression in hepatocytes and subsequently elevating its target genes including IL-6 and Lcn2, which are involved in the regulation of hepatic inflammation [5,60]. By using inhibitors of signal-transduction pathways, Otkjaer et al. discovered IL-1β induced IL-20 expression is dependent on p38 MAPK and NF-κB activity [57]. It is not difficult to find consistency in the downstream pathways of IL-1β and IL-20, which regulate the same target genes, and IL-1β mRNA is expressed at the same location as IL-20 mRNA [61,62]. Therefore. IL-1β is a key cytokine that affects IL-20 expression, and the IL-1β dependent signaling pathway may be an important molecular target for the regulation of IL-20 expression. IL-20 production is tightly regulated by body mechanisms, however, when this regulation becomes abnormal, excessive IL-20 production persists and leads to tissue damage. Importantly, a thorough investigation of IL-20 regulatory mechanisms is helpful in identifying IL-20 pathogenic targets in liver illnesses and proposing new treatment strategies.

## 5 Clinical applications of IL-20 in liver disease

**Fig. 3:**
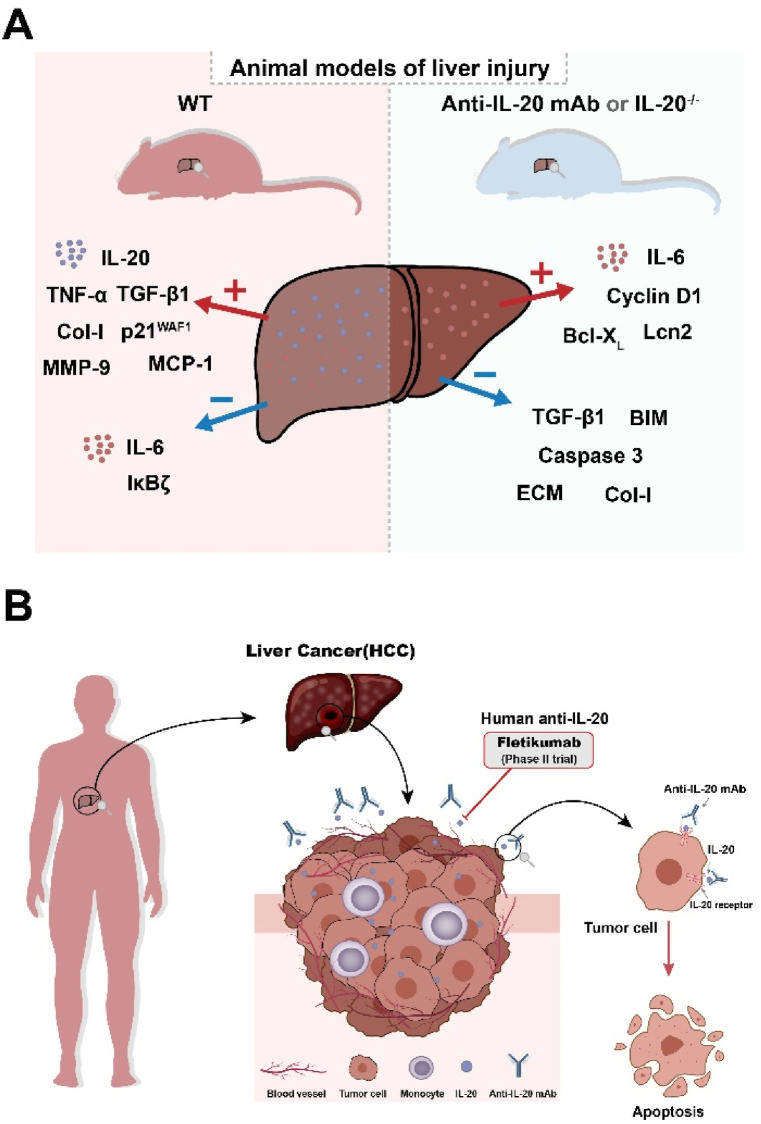
Function role of anti-IL-20 mAb or IL-20^−/−^ in liver diseases. (A) Anti-IL-20 mAb or IL-20^−/−^ regulates the expression of cytokines, genes and proteins in the liver. The use of anti-IL-20 reduces the inflammatory response, modulates immune cell function, and ameliorates damage in liver disease. (B) Anti-IL-20 monoclonal antibody can suppresses HCC progression. Fletikumab is a monoclonal antibody targeting to IL-20. Its efficacy, safety, tolerability, early signals of biologic and clinical effects were evaluated with patients (Clinical Trials.gov Identifier: NCT01636843, NCT01261767). TGF-β1, transforming growth factor-β1; MMP, matrix metalloproteinase; TNF-α, tumor necrosis factor-α; MCP-1, monocyte chemotactic protein-1; Col-I, type I collagen; p21^WAF1^, cyclin-dependent kinase inhibitor; Lcn2, lipocalin-2; ECM, Extracellular matrix; BIM, BCL-like 11; BCL-X_L_, B-cell lymphoma-extra large.

Mouse models have long been said to be instructive in the clinical application of disease [63], and IL-20-deficient mice are frequently used to illustrate the pro-inflammatory role of IL-20 in liver disease. In 1975, Kohler and Milstein ushered in the creation of hybridoma technology in the era of monoclonal antibodies (mAbs) [64]. mAbs are utilized in targeted therapeutic regimens for cancers, transplant rejection, autoimmune and infectious diseases, and a variety of other novel indications [65]. As anticipated, in animal models of liver injury, the research findings support the protective potential of anti-IL-20 mAb or IL-20 knockout (IL-20^−/−^) mice against liver injury (Fig. 3A).In a mice model of acute liver injury induced by Concanavalin A, serum IL-20 levels in the liver were significantly elevated. In contrast, IL-20^−/−^ mice exhibited reduced levels of alanine aminotransferase and aspartate aminotransferase as well as hepatic necrosis and increased levels of IL-6 after Concanavalin A administration compared to WT mice, suggesting that IL-20^−/−^ mice are resistant to Concanavalin A-induced hepatitis and are associated with selective upregulation of IL-6 with hepatoprotective properties. IL-6 is overexpressed in the liver tissue of IL-20^−/−^ mice and the hepatic expression of cyclin D1 and BCL-X_L_ was higher, while the expression of pro-apoptosis proteins BIM and cleaved caspase 3 was lower in Concanavalin A-treated IL-20^−/−^ mice. A mouse model of bacterial infection caused by Klebsiella pneumoniae (K.P.) showed elevated serum levels of IL-20 and reduced expression of IL-6 and several IκBζ-target genes in hepatocytes, including Lcn2, Ccl2, Ccl20, Csf3, S100a8 and S100a9. IL-20^−/−^ mice had lower mortality and lower circulating bacterial load than WT mice [66]. In CCL_4_-induced liver injury in mice, IL-20 was significantly elevated in mouse serum, upregulated the expression of TGF-β1 and p21^WAF1^, and resulted in cell cycle arrest in the Clone-9 rat hepatocyte cell line. Compared to WT mice, anti-IL-20 mAb and IL-20R mAb significantly inhibited TGF-β1 mRNA activity and attenuated CCL_4_-induced cellular damage [30]. Notably, the anti-IL-20 mAb suppressed the progression of HCC (Fig. 3B). Ding et al. confirmed that IL-20 promotes the development of HCC, and anti-IL-20 mAb attenuated the effects of IL-20 and inhibited liver tumorigenesis in a xenograft tumor nude mouse model [38]. Chiu et al. found that an anti-IL-20 mAb inhibited tumor growth in ML-1 cell-injected mice and suppressed the expression of TNF-α, cyclin D1, and MMP-9 in mice, suggesting that an anti-IL-20 mAb has the potential to be an effective therapeutic agent for HCC [39]. However, humanized antibodies targeting IL-20 are key to addressing the practical needs of clinical applications [67]. Fortunately, Fletikumab (NNC0109-0012), sponsored by Novo Nordisk A/S, is the first human monoclonal immunoglobulin G4 (IgG4) antibody to be considered for the treatment of psoriasis, rheumatoid arthritis, and other inflammatory diseases [68]. The efficacy, safety, tolerability, and early signals of biologic and clinical effects were evaluated in patients with rheumatoid arthritis and psoriasis (Clinical Trials.gov Identifier: NCT01636843 [69], NCT01261767 [70]). Promising results have been achieved in rheumatoid arthritis, and toxicology studies have not revealed any safety concerns [71]. It was well tolerated by patients with psoriasis, and no dose-limiting toxicity was observed. In addition, Fletikumab showed pharmacokinetics similar to those of other mAbs in both diseases [72]. Therefore, Fletikumab is being investigated as a potential drug for the treatment of inflammatory diseases [73]. In summary, these data indicate that anti-IL-20 may modulate immune system function by interfering with the expression of IL-20 and inflammatory mediators, thereby reducing the degree of inflammation and associated symptoms. However, further confirmation of its effectiveness is required through additional clinical trials. As more works are conducted in the near future, we expect to obtain more evidence to justify the treatment of anti-IL-20 mAb in liver disease.

## 6 Clinical implications of IL-20 in other organs

To demonstrate the therapeutic potential of IL-20, we enumerated the current clinical studies related to IL-20 to understand the functions of IL-20 in different diseases and to establish a foundation for developing the potential functions of IL-20 in liver diseases [74]. A complete list of all data sources is provided in Table 2. For example, obstructive sleep apnea (OSA) and NAFLD are chronic diseases with different clusters of associated co-morbidities that impact individual prognosis. Gaucher et al. used an unbiased multi-omics approach to reveal the molecular features that link NASH and OSA. They identified the dysregulation marker IL-20 that affects hepatic and extrahepatic organs and contributes to the development of metabolic and cardiovascular complications associated with OSA [22]. In mouse models of pancreatic ductal adenocarcinoma, there was concordance between IL-20 and HCC pathology status, associated with increased tumor fibrosis, programmed cell death 1 ligand 1 expression, and decreased overall survival [45]. Lu et al. analyzed IL-20 expression in tumor tissues of 72 patients with pancreatic ductal adenocarcinoma and found that overall survival was significantly influenced by IL-20 expression, with higher IL-20 levels predicting lower survival. In their previous studies, they similarly found that IL-20 promoted tumor growth in HCC, breast and oral cancer, and anti-IL-20 mAb treatment in a mouse model of cancer effectively mitigated the release of inflammatory mediators, inhibited tumor growth, and improved survival rates. This indicates that IL-20 released by inflammation at the early stages of tumor development promotes mutagenesis of cancer cells and accelerates their transformation to a highly malignant state. Antagonizing IL-20 overexpression in the tumor microenvironment effectively reduces the close association between inflammation and cancer [[75], [76], [77]]. One research measured IL-20 levels in the peripheral plasma of 83 patients with spondyloarthritis (SpA) and healthy controls. Compared with healthy controls, the plasma levels of IL-20 in patients with SpA increased by 57 %. This suggests that IL-20 mediates part of the inflammatory response of the patient, both in the liver and spine, and IL-20 levels increase with increasing disease severity and could be used clinically as an indicator of poor patient prognosis [78]. Tanay et al. suggested that IL-20 may play a role in the active phase of eosinophilic esophagitis (EoE). Patients with EoE have significantly elevated serum and esophageal levels of IL-20, and combining IL-20 with corticosteroids has been effective in reducing IL-20 expression in patients. Thus, inhibiting IL-20 expression not only by antagonizing itself or its receptor, but also by using exogenous drugs such as hormones in combination, opens new avenues for the treatment of IL-20 in liver disease [59]. Studying the potential application of IL-20 in diseases can be performed in preclinical animal models in addition to obtaining genetic data on serum, urine and cytokines in the clinical setting to determine the basis for which IL-20 can be used as a potential target. Hsu et al. found that IL-20 and its receptor IL-20R1 were overexpressed in the kidneys of streptozotocin-induced diabetic mice and induced apoptosis by activating the Caspase-8 pathway. In addition, kidney damage was ameliorated in mice with knocked out IL-20R1 receptor. This suggests that IL-20-mediated inflammatory responses are established in the presence of IL-20R1 [79]. Lee et al. found that IL-20 may regulate astrocyte activation and cause secondary damage in spinal cord injury (SCI) [80]. In a mouse model of high obesity, anti-IL-20 mAb therapy improved glucose tolerance and reduced the number of local inflammatory cells and macrophages in adipose tissue. This indicates a significant role of IL-20 in obesity-induced inflammation, which may be a useful treatment option for obese patients with metabolic disorders [81]. Altogether, a multitude of reports have found that IL-20 can be applied in different diseases, some of which are currently undergoing clinical trials with some positive preliminary data. Different disease models have highlighted the potential of IL-20 as a clinical treatment option. Similar to liver diseases, IL-20 behaves as a pro-inflammatory cytokine in most diseases and is involved in disease progression. For example, it inhibits apoptosis, promotes tumor growth and metastasis, and reduces survival. Antagonizing the release of IL-20 can effectively reduce local inflammation and cellular damage. Thus, IL-20 has the potential to serve as a biomarker in future clinical applications.

**Table 2:** Functional roles of IL-20 in other organs.

IL-20	Organs	Diseases	Functional roles of IL-20	[Ref]
IL-20	Nose and Throat	OSA	IL-20 is a dysregulated marker in the context of OSA and NASH	[22]
	Esophagus	EOE	IL- 20 interferes with the integrity of the esophageal epithelial barrier	[59]
	Eye	DED	IL-20 induces cell death in corneal epithelial cells	[102]
	Pancreas	PDAC	IL-20 is a critical mediator in PDAC progression.	[75]
	Spinal cord	SCI	IL-20 regulates astrocyte reactivation and axonal regeneration	[80]
	Kidney	Diabetic nephropathy	IL-20 induces cell apoptosis of podocytes	[79]
	Spine	SpA	IL-20 induces mineralization of human osteoblasts	[78]
	Skin	PsA	IL-20 induces the production of IL-6, IL-8 and MCP-1 by fibroblast-like synoviocytes	[103]
	Lung	NSCLC	IL-20 possesses anti-angiogenic properties within the NSCLC setting and is significantly up regulated by HDACi	[104]

OSA, Obstructive sleep apnea; EOE, Eosinophilic oesophagitis; DED, Dry eye disease; PDAC, Pancreatic ductal adenocarcinoma; SCI, Spinal cord injury; SpA, Spondyloarthritis; PsA, Psoriatic arthritis; NSCLC, Non-small cell lung cancer; NASH, Nonalcoholic steatohepatitis; MCP-1, Monocyte chemoattractant protein-1.

## 7 Future perspective

The biological function of IL-20 in liver disease has become clearer as more intensive studies are being conducted. Several studies have shown that IL-20 is aberrantly regulated in NAFLD, HBV, HCC, and liver fibrosis. Targeted studies and clinical applications of IL-20 are currently attracting considerable attention in the scientific field. In this section, we comprehensively explore potential new applications of IL-20 based on the latest technology and research findings.

Recently, genome editing technologies have provided important applications for the study of gene function in plants and animals. Clustered Regularly Interspaced Short Palindromic Repeats (CRISPR)-Cas9 are versatile genome editing tools that can be used in a variety of cell types and organisms. They can be used in genome-wide screens to study basic biological functions or to discover and validate potential drug targets for complex diseases [82]. For example, in previous studies, IL-20R1 was found to be a key factor in preventing transabdominal metastasis of ovarian cancer (OC), and by in vivo genomic CRISPR/Cas9 screening, when OC cells spread to the peritoneal cavity, they induced peritoneal mesothelial cells to express IL-20. Activation of IL-20RA downstream signaling in OC cells promotes macrophage polarization to M1 like subtypes and clear cancer cells [83]. Gene editing has been validated to explore the mechanism of IL-20 action in OC. Similarly, genome-wide screening using CRISPR-Cas9 can further explore the relevant signaling pathways and regulation of IL-20 in liver disease. Using this approach, we will be able to open up new possibilities for the treatment of liver diseases.

Nanomedicine is a class of nano-sciences for the detection and treatment of diseases, and has become an important development in modern medicine [37,84]. The ability to use nanoparticles to deliver drugs to specific cells for the detection and treatment of many diseases has been successfully developed and applied. Xie et al. developed and applied a nanocarrier with an IL-10 plasmid for safe and efficient tissue repair in a muscle injury model, and the sustained production of IL-10 by the nanocarrier greatly reduced splenomegaly and renal inflammation, with a positive safety profile in vivo [85]. Lee et al. developed an siRNA delivery system based on poly (lactic acid-glycolic acid) nanoparticles to promote skin absorption for topical psoriasis treatment. The nanocarriers showed less cytotoxicity and easy cellular uptake with reduced IL-6 expression [86]. Thus, it is possible to consider that the application of nanocarrier systems antagonizing IL-20 is both safe and reduces disease damage, providing new ideas for the prevention and treatment of liver disease by IL-20.

Many aspects contribute to liver inflammation, and inflammasomes are key to inflammation and innate immunity. Inflammasomes are multiprotein scaffolds activated by molecules associated with harmful signaling pathogens [87]. It assembles with the effector protein caspase-1 to form an intracellular multiprotein complex that promotes the secretion of cytokines, while inducing a state of cell death known as pyrolysis [88]. And the anti- or pro-inflammatory actions of cytokines in liver illnesses have a direct impact on how inflammasomes are stimulated [89]. In the case of IL-20, the main effect of inflammasome activation on liver diseases is unclear, and the development of IL-20 as a signaling marker of inflammasome activation is of enlightening importance for the control of liver diseases.

In summary, IL-20 may not only provide a novel link between basic life science and clinical medical research, but may also be applied in conjunction with a wide range of other cutting-edge research results, representing a research direction that is worthy of in-depth exploration.

## 8 Summary

**Fig. 4:**
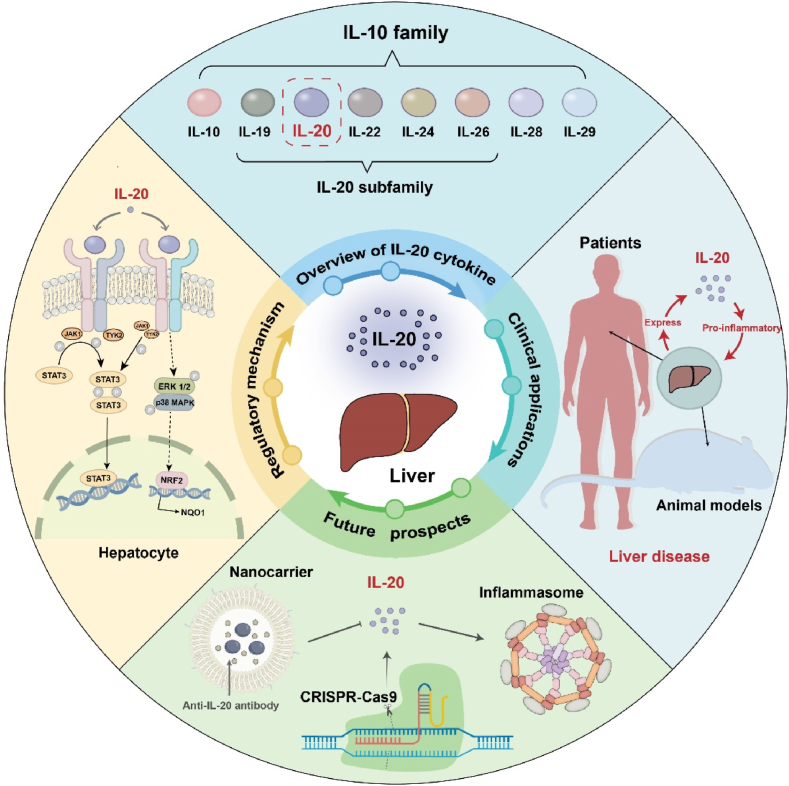
The role of IL-20 in liver diseases. IL-20 is involved in the regulation of inflammatory response, immune response and cell proliferation. We systematically reviewed the mechanism of action and clinical applications of IL-20 as a pro-inflammatory factor in different liver diseases. Targeted antagonistic therapy against IL-20 may be a new approach for the treatment of liver disease.

IL-20 acts as a potential target for liver diseases by combining its own receptors and signaling pathways to play a series of biofunctional regulatory roles in viral hepatitis, NAFLD, HCC and liver fibrosis. Insights into IL-20 in specific tissue and disease contexts may help develop new strategies for the treatment of liver diseases. To date, a range of IL-20 and IL-20 receptor antagonists have been developed, which have a wide range of clinical applications aimed at producing protective effects against tissue damage and inflammation. In conclusion, IL-20 is closely associated with liver diseases and has significant potential for the treatment of liver diseases (Fig. 4).

## Funding

This work was supported by the 10.13039/501100001809National Natural Science Foundation of China (82373932, 82172858), 10.13039/501100003995Natural Science Foundation of Anhui Province (2208085MH203), Anhui Translational Medicine Research Institute Project (2022-zhyx-C09), Anhui Outstanding Young Teachers Cultivation Program (YQZD2023023), Anhui Province Clinical Medical Research Translation Special Program (202204295107020023).

## Data availability statement

No data was used for the research described in the article.

## CRediT authorship contribution statement

**Kun Wang:** Writing – original draft. **He-Qin Zhan:** Writing – original draft. **Ying Hu:** Writing – review & editing. **Zhan-Yuan Yuan:** Writing – review & editing. **Jun-Fa Yang:** Writing – review & editing. **Da-Shuai Yang:** Writing – review & editing. **Liang-Song Tao:** Writing – review & editing. **Tao Xu:** Writing – review & editing.

## Declaration of competing interest

The authors declare that they have no known competing financial interests or personal relationships that could have appeared to influence the work reported in this paper.
